# Persistent phrenic nerve palsy after atrial fibrillation ablation: Follow‐up data from The Netherlands Heart Registration

**DOI:** 10.1111/jce.15368

**Published:** 2022-01-28

**Authors:** Daniel Mol, Lisanne Renskers, Jippe C. Balt, Rohit E. Bhagwandien, Yuri Blaauw, Vincent J. H. M. van Driel, Antoine H. G. Driessen, Arif Elvan, Richard Folkeringa, Rutger J. Hassink, Bart Hooft van Huysduynen, Justin G. L. M. Luermans, Jeroen Y. Stevenhagen, Pepijn H. van der Voort, Sjoerd W. Westra, Joris R. de Groot, Jonas S. S. G. de Jong, The Netherlands Heart Registration Ablation Committee

**Affiliations:** ^1^ Department of Cardiology OLVG Amsterdam The Netherlands; ^2^ Department of Cardiology and Cardiac Surgery, Amsterdam University Medical Centres University of Amsterdam Amsterdam The Netherlands; ^3^ Netherlands Heart Registration Utrecht The Netherlands; ^4^ Department of Cardiology St. Antonius Nieuwegein The Netherlands; ^5^ Department of Cardiology Erasmus Medical Centre Rotterdam The Netherlands; ^6^ Department of Cardiology University Medical Centre Groningen Groningen The Netherlands; ^7^ Department of Cardiology Haga Hospital Den Haag The Netherlands; ^8^ Department of Cardiology Isala Zwolle The Netherlands; ^9^ Department of Cardiology Medical Centre Leeuwarden Leeuwarden The Netherlands; ^10^ Department of Cardiology University Medical Centre Utrecht Utrecht The Netherlands; ^11^ Department of Cardiology Amphia Breda The Netherlands; ^12^ Department of Cardiology Maastricht University Medical Centre Maastricht The Netherlands; ^13^ Department of Cardiology Medisch Spectrum Twente Enschede The Netherlands; ^14^ Department of Cardiology and Cardiac Surgery Catharina Hospital Eindhoven The Netherlands; ^15^ Department of Cardiology Radboud University Medical Centre Nijmegen The Netherlands

**Keywords:** ablation, atrial fibrillation, conventional RF, cryoballoon, phased RF, phrenic nerve palsy, thoracoscopic ablation

## Abstract

**Background:**

Persistent phrenic nerve palsy (PNP) is an established complication of atrial fibrillation (AF) ablation, especially during cryoballoon and thoracoscopic ablation. Data on persistent PNP reversibility is limited because most patients recover <24 h. This study aims to investigate persistent PNP recovery, freedom of PNP‐related symptoms after AF ablation and identify baseline variables associated with the occurrence and early PNP recovery in a large nationwide registry study.

**Methods:**

In this study, we used data from the Netherlands Heart Registration, comprising data from 9549 catheter and thoracoscopic AF ablations performed in 2016 and 2017. PNP data was available of 7433 procedures, and additional follow‐up data were collected for patients who developed persistent PNP.

**Results:**

Overall, the mean age was 62 ± 10 years, and 67.7% were male. Fifty‐four (0.7%) patients developed persistent PNP and follow‐up was available in 44 (81.5%) patients. PNP incidence was 0.07%, 0.29%, 1.41%, and 1.25%, respectively for patients treated with conventional‐RF, phased‐RF, cryoballoon, and thoracoscopic ablation respectively. Seventy‐one percent of the patients fully recovered, and 86% were free of PNP‐related symptoms after a median follow‐up of 203 (113–351) and 184 (82–359) days, respectively. Female sex, cryoballoon, and thoracoscopic ablation were associated with a higher risk to develop PNP. Patients with PNP recovering ≤180 days had a larger left atrium volume index than those with late or no recovery.

**Conclusion:**

After AF ablation, persistent PNP recovers in the majority of patients, and most are free of symptoms. Female patients and patients treated with cryoballoon or thoracoscopic ablation are more prone to develop PNP.

## 1 INTRODUCTION

Phrenic nerve palsy (PNP) is a common complication of atrial fibrillation (AF) ablation. Persistent PNP (lasting > 24 h) occurred in 1.5% of the patients who underwent cryoballoon (CB) ablation in the Netherlands.^1^ PNP frequently complicates CB ablation, but has also been described after radiofrequency (RF), phased RF, or thoracoscopic ablation.^1^, ^2^, ^3^, ^4^, ^5^, ^6^, ^7^ Most PN injuries recover during the initial hospital admission. However, longer‐lasting PNP resulting in unilateral diaphragm paralysis can result in exercise intolerance, shortness of breath, or orthopnea.^8^

Persistent PNP after AF ablation is a well‐known complication, but data on its reversibility is sparse or limited to CB ablation.^3^, ^4^, ^5^, ^6^, ^9^ We investigated rates of PNP‐related symptoms and PNP recovery, and identified baseline variables associated with persistent PNP after AF ablation.

## 2 METHODS

We included all patients who underwent AF ablation in 2016 or 2017 from the Netherlands Heart Registration (NHR), a nationwide quality registry in which 14 out of 16 Dutch ablation centers report outcomes of AF ablation.^1^ We performed additional follow‐up in patients with persistent PNP. During CB ablation, phrenic nerve pacing from the superior vena cava was performed to monitor the phrenic nerve function and all patients received a chest X‐ray after surgical ablation. Follow‐up data were collected in a cross‐sectional manner according to standard clinical care and based on physicians’ discretion. Besides the patients’ history, follow‐up of patients with PNP consisted of a chest X‐ray, sniff test, and/or physical examination. Patients were considered to have proven PNP if diaphragm elevation was present on a chest X‐ray following AF ablation. Persistent PNP was defined as PNP lasting >24 h.^1^ At follow‐up, PNP recovery was defined as normalization of abnormalities at sniff test, chest X‐ray, and/or as specified in the medical chart. A waiver for informed consent for participation in the NHR was previously obtained from the Ethics Committee MEC‐U, Nieuwegein, The Netherlands.

The primary outcome was recovery of a proven PNP after AF ablation. Secondary outcomes included: the presence of PNP‐related symptoms and early (≤180 days) or late/no (>180 days) PNP recovery. We further sought to identify baseline clinical variables associated with PNP occurrence.

Normally distributed clinical variables are presented with a mean ± standard deviation, non‐normally distributed with a median and interquartile range (IQR), and categorical variables with numbers and percentages. Parametric *t*‐test, nonparametric Mann–Whitney *U* test, *χ* ^2^ test, and Fishers’ exact test were used to compare groups. The endpoints proven PNP recovery and freedom of PNP‐related symptoms are presented in survival curves. Multivariate logistic regression analysis was performed for adjustment for co‐variables with a univariate *p*‐value < .1. Data are presented as adjusted odds ratio (OR) and 95%‐confidence intervals (CI). R‐studio (version 1.1.383) was used for statistical analysis.

## 3 RESULTS

The data set comprised 9549 procedures in 8498 patients. PNP data was available from 7433 (78%) procedures in 7026 (83%) patients. The mean age was 62 ± 10 years, and 68% of patients were male (Table 1). AF ablation was performed with C‐RF (41.1%), Ph‐RF (also including multiarray septal catheter and multi‐array ablation catheter [MAAC/MASC]) (9.5%), CB (39.6%), LB (0.07%), or thoracoscopic (also including hybrid ablation) (9.8%).

**Table 1:** Baseline characteristics

	Overall (*n* = 7433)	No PNP (*n* = 7379)	PNP (*n* = 54)	*p* value PNP yes/no
Male	5035 (67.7)	5013 (67.9)	22 (40.7)	<.001
Age (year)	61.6 ± 9.6	61.6 ± 9.6	61.8 ± 9.8	.862
BMI (kg/m^2^)	(*n* = 7372)	(*n* = 7318)		.753
	27.4 ± 4.2	27.4 ± 4.2	27.6 ± 3.7	
Height (cm)	(*n* = 7390)	(*n* = 7336)		<0.001
	178.9 ± 10.0	179.0 ± 10.0	174.0 ± 10.0	
LAVI (ml/m^2^)	(*n* = 3964) 36.6 ± 11.8	(*n* = 3933) 36.6 ± 11.8	(*n* = 31) 39.8 ± 13.5	.200
Mitral valve regurgitation	(*n* = 6293)	(*n* = 6243)	(*n* = 50)	.600
None/mild	5886 (93.5)	5840 (93.6)	46 (92.0)	
Moderate	393 (6.3)	389 (6.2)	4 (8.0)	
Severe	14 (0.2)	14 (0.2)	0 (0.0)	
CHA_2_DS_2_VASc	(*n* = 7242) 1.6 ± 1.4	(*n* = 7289) 1.6 ± 1.4	(*n* = 53) 1.9 ± 1.4	.102
Type AF	(*n* = 7352)	(*n* = 7299)	(*n* = 53)	.736
Paroxysmal	5044 (68.6)	5004 (68.6)	40 (75.5)	
Persistent	2083 (28.3)	2,071 (28.4)	12 (22.6)	
Longstanding persistent	189 (2.6)	188 (2.6)	1 (1.9)	
Other atrial arrhythmia	36 (0.5)	36 (0,5)	0 (0.0)	
Ablation method	(*n* = 7,362)	(*n* = 7,308)		<.001
C‐RF	3,028 (41.1)	3,026 (41.4)	2 (3.4)	
Phased‐ RF incl. MASC/MAAC	697 (9.5)	695 (9.5)	2 (3.7)	
Cryoballoon	2,909 (39.6)	2,868 (39.2)	41 (75.9)	
Laser balloon	5 (0.07)	5 (0.07)	0 (0.0)	
Thoraco‐scopic incl. hybrid ablation	723 (9.8)	714 (9.8)	9 (16.7)	
Previous LA ablation	(*n* = 7411) 1630 (22.0)	(*n* = 7358) 1621 (22.0)	(*n* = 53) 9 (17.0)	.473

*Note*: Baseline characteristics and group comparison. Mean standard deviation (±), number (%).

Abbreviations: AF, atrial fibrillationBMI, body mass index; CHA2DS2 VASc, congestive heart failure, hypertension, age (≥75, doubled), diabetes, stroke (doubled), vascular disease, age (≥ 65), sex; C‐RF, conventional radiofrequency; LA, left atrium; LAVI, left atrial volume index; MAAC, multi‐array ablation catheter; MASC, multi‐array septal catheter; PNP, phrenic nerve palsy.

Following AF ablation, 54 (0.7%) patients developed proven PNP lasting >24 h. Of those, follow‐up data were available in 44 (81.5%) patients. All 44 patients had follow‐up data on PNP‐related symptoms and objective follow‐up data on the persistence of PNP was available in 41 (76.0%) patients.

**Figure 1:**
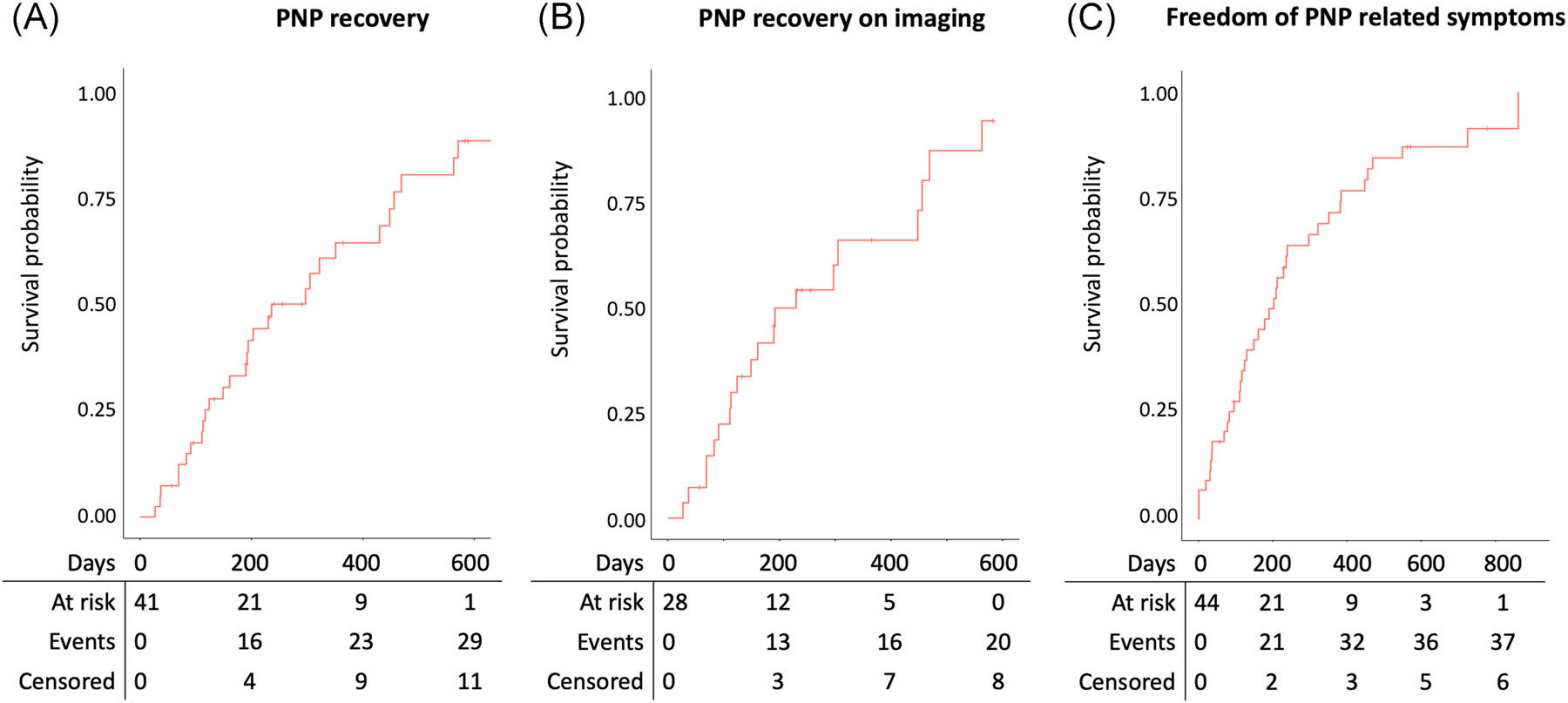
Survival analysis. (A) survival analysis of phrenic nerve palsy (PNP) recovery based on imaging techniques and physicians’ reports. (B) PNP recovery of patients in whom imaging techniques were used on final follow‐up. (C) Freedom of PNP‐related symptoms

**Figure 2:**
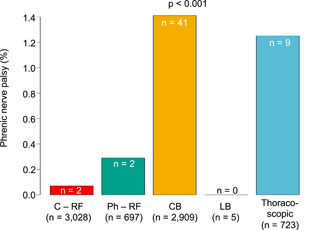
Phrenic nerve palsy. The occurrence of phrenic nerve palsy in percentage among the ablation modalities; conventional radiofrequency (C – RF), phased – RF (Ph – RF), cryoballoon (CB), laser balloon (LB), and thoracoscopic ablation

Of the 41 patients with follow‐up data on the persistence of PNP, imaging techniques at final follow‐up were used in 28/41 (68.3%) patients. In the other 13 (31.7%) patients, physicians’ reports explicitly stated that PNP had recovered. PNP fully recovered in 29/41(70.7%) patients after a median follow‐up of 203 (113–351) days (Figure 1A), confirmed with imaging techniques in 20/28 (71.4%) patients after a median follow‐up of 190 (106–299) days (Figure 1B). Among the 44 patients with follow‐up data on PNP‐related symptoms, 38 (86.4%) were free of PNP‐related symptoms after a median follow‐up of 184 (82–359) days (Figure 1C). Notably, the three patients in whom functional data on PNP persistence was not available were all free of PNP‐related symptoms. Of the patients with PNP, 59.3% were female compared with 32.1% of the patients without PNP (*p* < .001). Patients with PNP had a significantly shorter stature than patients without PNP (174 ± 10 cm vs. 179.0 ± 10 cm, *p* < .001). PNP occurred in 0.07% of patients treated with C‐RF, 0.29% of patients treated with Ph‐RF MAAC/MASC, 1.41% of CB, and 1.25% of thoracoscopic ablation (*p* < .001, Figure 2). No PNP occurred in the LB group (*n* = 5) (Table 1). Multivariate analysis demonstrated that, compared with C‐RF, CB and thoracoscopic ablation were associated with an increased risk for PNP (OR 21.12, CI 6.49–129.75 and 18.93, CI 4.86–124.41, respectively) (Table 2). In addition, female sex was independently associated with an increased risk of PNP (OR 2.32, CI 1.07–5.06) (Table 2).

**Table 2:** Multivariate regression analysis of baseline variables associated with the occurrence of phrenic nerve palsy

	Odds ratio	95% confidence interval	*p* value
Female sex	2.32	1.07–5.06	.034
Height	0.98	0.95–1.02	.374
Ablation method			
Conventional‐RF	ref		
Phased‐ RF incl. MASC/MAAC	4.40	0.53–36.78	0.139
Laser balloon	NA		
Cryoballoon	21.12	6.49–129.75	<.001
Thoraco‐scopic incl. hybrid ablation	18.93	4.86–124.41	<.001

Abbreviations: MAAC, multi‐array ablation catheter; MASC, multi‐array septal catheter; RF, radiofrequency.

Of the 41 patients with proven PNP, PNP recovered in 16/41 (39%) ≤180 days, in 13/41 (32%) after >180 days, and in 12/41 (29%) no recovery was documented. Patients who recovered ≤180 days had a larger LAVI than the patients with late or no documented PNP recovery (45.6 ± 10.3) versus 34.4 ± 11.9) ml/m^2^, *p* = .018). Amongst the ablation modalities, 13/31 (41.9%) patients treated CB and 3/7 (42.9%) patients treated with thoracoscopic ablation PNP recovered ≤180 days. All patients treated with C‐RF (*n* = 2) and Ph‐RF (*n* = 1) recovered after 180 days and we observed no recovery in 2/7 (28.6%) patients after thoracoscopic ablation and in 10/31 (32.3%) patients after CB (*p* = .820).

## 4 DISCUSSION

This is the largest study on the clinical course of persistent PNP in patients who underwent AF ablation with five different ablation modalities.

PNP fully recovered in 71% of the patients and 86% were free of PNP‐related symptoms. These findings are in line with other studies reporting phrenic nerve recovery in 78%–100% of the patients.^3^, ^4^, ^5^, ^9^ After thoracoscopic ablation, PNP has been described in up to 11% of the patients, of whom 80% recovers within 12 months of follow‐up.^6^ In contrast to catheter ablation, PNP during thoracoscopic ablation can also occur after a blunt trauma from manipulation of ablation and endoscopic tools or traction on the pericardial cradles.^7^, ^10^

An essential factor for PNP occurrence during catheter ablation is the distance between the ablation site and the phrenic nerve. Smaller and more distally positioned CB has been associated with more PNP.^11^, ^12^ Also, an early study reporting PNP after radiofrequency ablation demonstrated that most patients who developed PNP received the more distal segmental or focal pulmonary vein isolation.^13^ In contrast, the FIRE and ICE trials did not observe any PNP in patients who underwent PV atrium radiofrequency ablation.^14^ This suggests a lower risk for PNP because most energy during conventional RF ablation is delivered at the antrum of the pulmonary vein.

Aside from the distance between the phrenic nerve and the ablation site, the second‐generation CB with improved cooling abilities has increased the number of patients developing PNP.^15^ Also, PNP in patients treated with the second generation CB appears to recover slower than in patients treated with the first generation CB. Similarly, PNP after LB ablation was associated with a longer recovery time compared to CB.^4^, ^5^ Here, we did not observe any significant difference in recovery time among the ablation modalities.

We show that female patients have a 2.3 times higher risk for persistent PNP. Compared with males, females tend to have a smaller left atrium, and thinner atrial wall thickness.^16^, ^17^, ^18^ Also, the right phrenic nerve is located more anteriorly in the thoracic cavity in females than in males.^19^ A small study investigating 28 human cadavers demonstrated that the distance between right superior pulmonary vein – phrenic nerve was smaller than 10 mm in 67% of the females compared to 53% of males.^20^ Additionally, the authors also observed a trend towards an increased pulmonary vein – phrenic nerve distance with an increasing left atrial size.^20^ These anatomical differences could potentially increase the risk of collateral phrenic nerve damage in female patients.

This study has some limitations: The number of PNP described are the reported PNP, because the data from this study is based on a nationwide registry study, there are missing variables and PNP data, both on the presence as on the absence of PNP, was not reported in 22% of the patients. In addition, follow‐up on PNP was not standardized but according to standard clinical care. Follow‐up data were not available in 10/54 patients with PNP. We presume that patients with more severe symptoms are monitored more intensively compared to asymptomatic patients. Monitoring of phrenic nerve integrity during ablation was rarely performed in patients ablated with other modalities than CB. This may have led to an under detection of asymptomatic PNP and disproportionally biased the study results towards higher PNP incidence in patients treated with CB as compared to the patients treated with the other catheter ablation modalities.

## 5 CONCLUSION

In this large real‐world study, PNP recovered in most patients after AF ablation and 86% of the patients were free of PNP‐related symptoms. Female patients and patients treated with CB or thoracoscopic ablation were at higher risk to develop PNP.

## AUTHOR CONTRIBUTIONS

All authors take responsibility for all aspects of the reliability and freedom from bias of the data presented and their discussed interpretation.

## Supporting information

Supporting information.

## Data Availability

The data underlying this article were provided by The Netherlands Heart Registration by the permission of the participating hospitals. Data are available upon reasonable request to the corresponding author and with permission of the Netherlands Heart Registration.
