# Supplementary material for: Persistent phrenic nerve palsy after atrial fibrillation ablation: Follow‐up data from The Netherlands Heart Registration
Source: J Cardiovasc Electrophysiol. 2022 Jan 28;33(3):559–64. doi: 10.1111/jce.15368 (PMC9303579; doi:10.1111/jce.15368)
Supplement: Supplementary file 1 — Supporting information. [file JCE-33-559-s001.docx]

**Supplementary data**

Members of the Netherlands Heart Registration ablation Committee:

J.C. Balt, St. Antonius, Department of Cardiology, Nieuwegein, the Netherlands.

R.E. Bhagwandien, Erasmus Medical Centre, Department of Cardiology, Rotterdam, the Netherlands.

Y. Blaauw, University Medical Centre Groningen, Department of Cardiology, Groningen, the Netherlands

V.J.H.M. van Driel, Haga hospital, Department of Cardiology, Den Haag, the Netherlands

A.H.G. Driessen, Amsterdam University Medical Centres/University of Amsterdam, Department of Cardiac Surgery, Amsterdam, the Netherlands

A. Elvan, Isala, Department of Cardiology, Zwolle, the Netherlands

R. Folkeringa, Medical Centre Leeuwarden, department of Cardiology, the Netherlands.

R.J. Hassink, University Medical Centre Utrecht, Department of Cardiology, Utrecht, the Netherlands.

B. Hooft van Huysduynen, Amphia, Department of Cardiology, Breda, the Netherlands

J.S.S.G. de Jong, OLVG, Department of Cardiology, Amsterdam, the Netherlands

M. Kemme, Amsterdam University Medical Centres, Department of Cardiology, Amsterdam, the Netherlands

J.G.L.M. Luermans, Maastricht University Medical Centre, Department of Cardiology, Maastricht, the Netherlands

Y.J. Stevenhagen, Medisch Spectrum Twente, Department of Cardiology, Enschede, the Netherlands

S.A.I.P. Trines, Leiden University Medical Centre, Department of Cardiology, Leiden, the Netherlands

P. van de Voort, Catharina, Department of Cardiology, Eindhoven, the Netherlands

Contact information:

E-mail: info@nederlandsehartregistratie.nl
